# Neutral Position or Contralateral Head Rotation in Vagus Nerve Stimulation Surgery: A Study of Surgical Pathway and Nervus Vagus Position with Peroperative Ultrasonography

**DOI:** 10.3390/brainsci15040385

**Published:** 2025-04-08

**Authors:** Güven Gürsoy, Gönül Güvenç

**Affiliations:** Neurosurgery Department, Faculty of Medicine, Muğla Sıtkı Koçman University, Muğla 48000, Turkey; drgonul35@gmail.com

**Keywords:** vagal nerve stimulation, peroperative ultrasonography, vagus nerve, sternocleidomastoid muscle

## Abstract

*Background and Objectives*: This study aimed to discuss positional changes in the sternocleidomastoid (SCM) muscle and vagus nerve with head position, their effect on the surgical path, positional variations, the selection of an appropriate position for surgery, their effects on the surgical procedure, and complications by using peroperative ultrasonography. *Materials and Methods*: Vagal nerve stimulation surgery patients over the age of 18 years were included. Peroperative ultrasonography images were scanned, and changes in head position and anatomical and positional variations in the SCM muscle and vagus nerve at the surgical incision level were examined. *Results*: SCM localization was most frequently observed in the lateral aspect of the carotid sheath (n:16) in neutral position, while it was mostly observed in the medial aspect of the carotid sheath (n:16) at a 15 degree rotation. The vagus nerve was mostly observed between the jugular vein and carotid artery in neutral position (n:21), and it was observed at the same position at a 15 degree rotation (n:17). The positional change of the SCM muscle with head position was found to be statistically significant (*p* < 0.001), while the positional change of the vagus nerve was not (*p*:0.198). *Conclusions:* The SCM muscle closes the surgical path with head rotation by either deviating over the carotid sheath or increasing its deviation. In addition to its anatomical variations, the vagus nerve shows different positional changes with head rotation. Deciding on the head position in vagal nerve stimulation surgery, using peroperative ultrasonography rather than a routine position, may be effective in reducing surgical time and possible complications.

## 1. Introduction

The use of vagal nerve stimulation has become increasingly common, especially in patients with drug-resistant epilepsy, since its use in humans was first reported in 1988 [1]. In patients who do not respond to medical treatment, especially if there is a single focus in a relatively safe area, curative surgery is preferred, and vagal nerve stimulation surgery comes to the forefront in generalized or multifocal epilepsy and unresectable epileptogenic focus cases [2]. Over the years, major depression has become an indication for vagal nerve stimulation [3]. Therefore, since its first use, the patient spectrum has expanded and the number of surgical cases has increased.

Vagal nerve stimulator implantation was described technically by Reid, and this description is still being used [4]. Although the left vagus is generally preferred because the right vagus stimulates the sinoatrial node and may result in bradycardia and asystole, the use of the right vagus has been reported in rare cases [5,6]. The head is fixed with extension, kept straight, or rotated 15 degrees towards the opposite side for surgery. The sternocleidomastoid (SCM) muscle is located on or medial to the carotid sheath. Passing through the medial aspect of the muscle, the vagus is located within the carotid sheath. Stabilizing anchor, positive, and negative electrodes are placed. After combining the battery and lead sections with a passer, the system is ready for use. In the meantime, an important step is to identify the vagal nerve, to avoid placing leads on the inferior (recurrent) laryngeal nerve, and to perform carotid artery and internal jugular vein traction as little and for as short a time as possible. Vagal nerve injury, hoarseness due to vocal cord paralysis, dyspnea, dysphagia, laryngopharyngeal dysfunction and neuralgia, cerebral ischemia, and venous infarcts are important complications in this step [7].

Although the cervical vagal nerve is expected to be located within the carotid sheath, between the common or internal carotid artery and the internal jugular vein, branching patterns can be seen within the sheath, and positional variations can be seen with the carotid artery and internal jugular vein [8].

The aim of this study is to determine the peroperative vagal nerve position and the location of the carotid artery, jugular vein, and SCM muscle in the surgical path using ultrasonography, which provides real-time imaging during vagal nerve stimulation surgery, and to discuss their effects and contributions to the surgical procedure to be performed and complications that may develop.

## 2. Materials and Methods

### 2.1. Study Design

This retrospective clinical study was designed to investigate the positional effects of the SCM muscle and nervus vagus in vagus nerve stimulation surgery, and participants were operated on at the Muğla Training and Research Hospital (affiliated with Muğla Sıtkı Koçman University) by the same surgeon between 2021 and 2024.

### 2.2. Study Population

The study included both male and female patients aged 18 years and older. Primary VNS implantation patients were included, whereas revision patients (battery or lead revision) and patients with a missing ultrasonographic image were excluded from the study.

### 2.3. Data Collection

Age and gender information was documented, and archives were searched for ultrasonography images. The position of the SCM muscle according to the carotid sheath and the vagus nerve within the carotid sheath was documented in the neutral position and at 15 degrees of rotation to the opposite (right) side.

### 2.4. Surgical and Radiological Procedures

**Figure 1:**
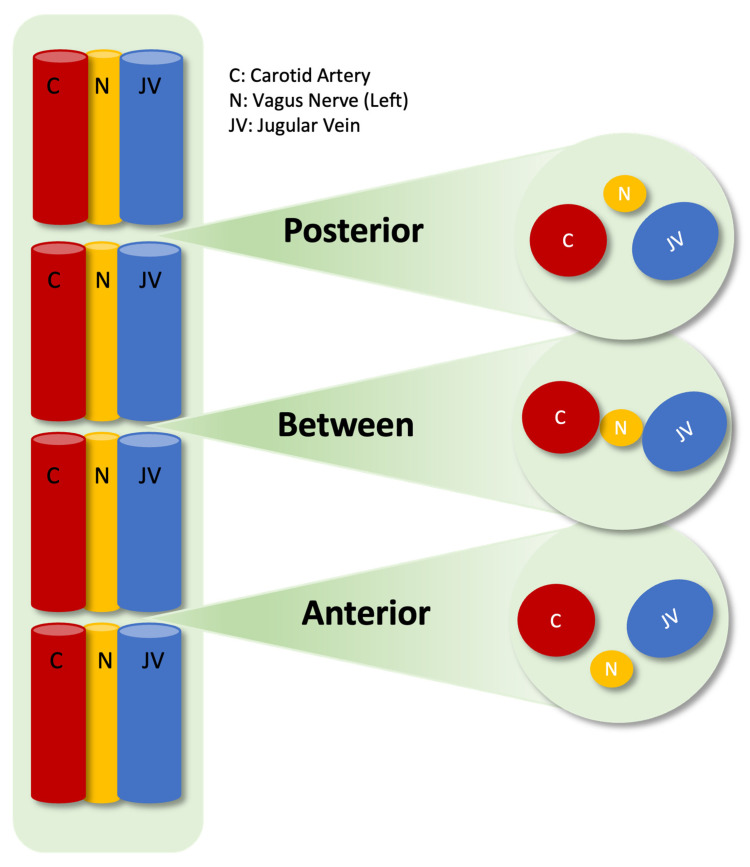
Positions of the left nervus vagus inside the left carotid sheath.

The incision point for vagal nerve stimulation is on the left side of the neck, mid-way between the chin and the sternal notch, with 1/3 of the incision lying medial to the SCM, and 2/3 remaining lateral to the SCM transversely. All studies were performed preoperatively, after intubation and head extension, by the same surgeon with an L12-5 MHz real-time linear array transducer ultrasound system: Philips, Bothell, WA, USA. All ultrasonographic images were obtained at this incision level. Using convex or linear probes, the positioning of the SCM muscle relative to the carotid sheath was first examined. The muscle is grouped as lateral to, above, or medial to the sheath. The carotid artery, jugular vein, and nervus vagus within the carotid sheath were visualized using transverse sections, and the positioning of the vagus nerve was examined. These images were taken in both neutral position and with head rotation. There are complex descriptions of localization stated in the literature for the nervus vagus, which include medial, dorsomedial, and dorsal to the jugular vein; lateral, ventral, or dorsal to the carotid artery; and their combinations [9]. The most effective grouping method that would facilitate surgical access was considered and examined by grouping according to the vascular structures within the carotid sheath. Structures were grouped as “anterior” for the nervus vagus located in front of the internal jugular vein or carotid artery, as “between” for the nervus vagus located between two vascular structures, and as “posterior” for the nervus vagus located behind either of the two vascular structures (Figure 1).

### 2.5. Statistical Analysis

All records and data regarding the patients were analyzed with the SPSS 23.00 statistical package program. The suitability of a normal distribution for the variables was examined by visual and analytical methods (Kolmogorov–Smirnov/Shapiro–Wilk tests). Descriptive analyses were given using the median and interquartile range for variables that were not normally distributed. From non-parametric data, the vagus nerve—SCM localization according to the neutral and 15-degree positions of the neck—was compared using the McNemar–Bowker test. Cases where the *p*-value was below 0.05 were considered statistically significant results.

## 3. Results

Forty-four patients who underwent vagal nerve stimulation surgery were identified between the specified dates. Five patients underwent battery revision surgery, and three patients’ ultrasonography images were not available, so eight patients were excluded from the study. Statistical analyses were evaluated for 36 patients. Overall, 55.6% (n:20) of the patients were female and 44.4% (n:16) were male, and the average age was 31.97 ± 5.54. When the changes in the SCM muscle and vagal nerve according to position were examined according to age, no statistically significant difference was detected. The distribution of the data according to gender groups is given in Table 1.

In 44.4% (n:16) of the patients, SCM localization was visualized lateral to the carotid sheath in the neutral position. It was visualized medial to the carotid sheath in 44.4% (n:16) of the patients when in a 15-degree rotation position. This positional change of the SCM muscle with head rotation is statistically significant (*p* < 0.001). The SCM muscle, which was located lateral to the carotid sheath in 16 patients in the neutral position, was displaced above the carotid sheath in 11 patients with head rotation and remained lateral to the carotid sheath in 5 patients. The SCM muscle, which was located above the carotid sheath in 15 patients in the neutral position, was displaced medial to the carotid sheath in 11 patients with head rotation and remained above the carotid sheath in 4 patients. It was observed that in 5 patients in neutral position, the SCM muscle located medial to the carotid sheath was not displaced by head rotation (Table 2).

Although the nerve vagus localization was visualized between the jugular vein and carotid artery in 58.3% (n:21) of the patients in neutral position and in 47.2% (n:17) in the 15-degree rotation position, no statistically significant difference was detected for the change in nervus vagus localization with head rotation (*p*:0.198). When looking at the localization change of the vagal nerve according to head position, the nervus vagus was located anterior to the vascular structures consisting of the carotid artery and jugular vein in three (8.3%) patients in the neutral position. The vagus nerve remained anterior to the vascular structures in two patients and was displaced between the vascular structures in one patient with head rotation. In the neutral position, it was located between the vascular structures in 21 (58.3%) patients, but with head rotation, it remained between the vascular structures in 10 patients, moved to the posterior of the vascular structures in 6 patients, and moved to the anterior of the vascular structures in 5 patients. It was observed that in the neutral position, it was located at the posterior of the vascular structures in 12 (33.3%) patients, and with head rotation, it moved between the vascular structures in 6 patients, remained posterior to the vascular structures without moving in 4 patients, and moved towards the anterior of the vascular structures in 2 patients (Table 3).

### 3.1. Case Presentation

#### 3.1.1. Contralateral Head Rotation for Nervus Vagus

**Figure 2:**
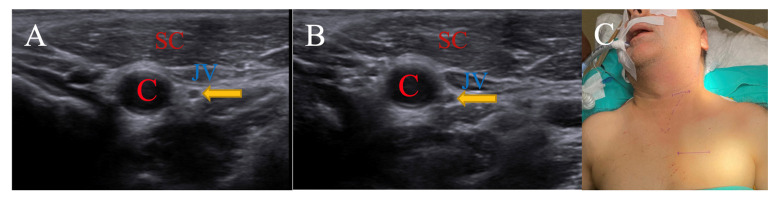
(**A**) is neutral position, while (**B**) is 15-degree right rotation. Patient is operated on in 15-degree right rotation position, as seen in (**C**). (C: carotid artery; JV: jugular vein; SC: sternocleidomastoid muscle; arrow: nervus vagus).

For a patient in their 40s, it was observed that the SCM muscle extended medially to the carotid sheath in the neutral position and did not change with 15 degrees of rotation in the peroperative USG images. It was seen that the n. vagus, while in a neutral position behind the jugular vein, changed position to between the carotid artery and jugular vein with a 15-degree rotation. Even though the SCM muscle did not change with position, the operation was performed with a 15-degree right rotation in order to access the vagal nerve more easily, reduce vascular retraction, and reduce the risk of complications due to all these factors. Due to the ability to reach the vagus nerve earlier and perform its dissection more easily compared to situations where it is located behind vascular structures, the surgery duration may be shortened by dozens of minutes (Figure 2).

#### 3.1.2. Neutral Position for Sternocleidomastoid Muscle

**Figure 3:**
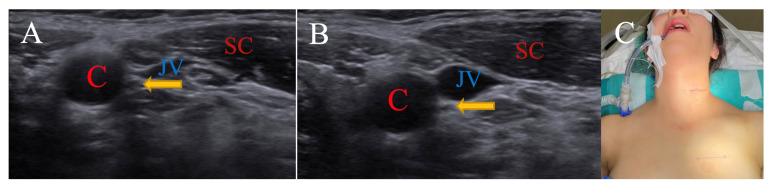
(**A**) is neutral position, while (**B**) is 15-degree right rotation. Patient is operated on in neutral position (**C**). (C: carotid artery; JV: jugular vein; SC: sternocleidomastoid muscle; arrow: Nervus vagus).

This is a patient around 30 years old. It was observed that the SCM muscle rolled over the carotid sheath with a 15-degree rotation while in a neutral position, lateral to the carotid sheath in peroperative USG images. The localization of the vagus nerve, which is located between the vascular structures in the neutral position, did not change with the head rotation. The patient was operated on in neutral position (Figure 3).

#### 3.1.3. Neutral Position for Nervus Vagus

**Figure 4:**
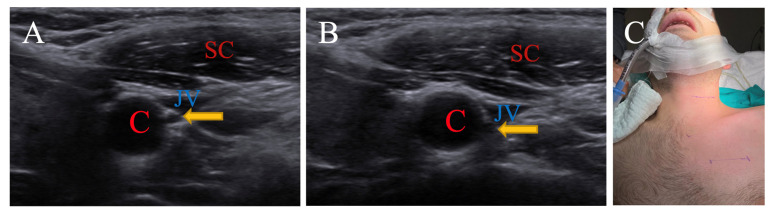
(**A**) is neutral position, while (**B**) is 15-degree right rotation. Patient is operated on in neutral position (**C**). (C: carotid artery; JV: jugular vein; SC: sternocleidomastoid muscle; Arrow: nervus vagus).

This case is an adult patient. The SCM muscle is medial to the carotid sheath in the neutral position, and this location does not change with a 15-degree right rotation. The n. vagus, on the other hand, remains between the carotid artery and jugular vein, both in a neutral position and with a 15-degree rotation, but is displaced slightly posteriorly. The patient was operated on in a neutral position for easier access (Figure 4).

## 4. Discussion

It has been determined that the sternocleidomastoid muscle, with a 15-degree rotation of the head, makes surgical access more difficult by covering the carotid sheath. Therefore, the neutral position may be more suitable in terms of surgical path. On the other hand, the vagus nerve may be found in different localizations with or without rotation due to its anatomical variations. Peroperative ultrasonography, which can be used for this reason, is simple, is usable for every patient, has very few side effects, can reveal additional pathologies, and is a cheap method that will have a positive effect on reducing complications. To the best of our knowledge, we are the first to discuss using peroperative ultrasonography for determining the head position in identifying the localization of the nervus vagus and sternocleidomastoid muscle in vagal nerve stimulation surgery. We are also the first to investigate the positional change of the vagus nerve and sternocleidomastoid muscle with rotation of the head.

Sadahiro et al. examined 23 patients who underwent vagal nerve surgery preoperatively, determined the place where the vagus nerve crosses the SCM muscle in the ultrasonographic imaging sections, classified 2 patients as high type, 5 patients as middle type, and 16 patients as low type, and touched upon the importance of variations in 2024 [10]. No article has been found in the literature about head rotation and the displacement of the SCM muscle relative to the carotid sheath. Our study is important in this respect. The peroperative ultrasonography we performed was carried out at the incision level appropriate for this surgical technique. It was observed that the SCM muscle, one of the structures on the surgical path, was located lateral to the carotid sheath in 16 patients when the head was in a neutral position, remained in the same place in 5 patients with a 15-degree rotation, and was displaced above the carotid sheath in 11 patients. Similarly, it was observed that the SCM muscle, located above the carotid sheath in the neutral position of the head, remained the same in 4 patients with a 15-degree rotation and was displaced medial to the carotid sheath in 11 patients. It was also seen that the SCM muscle, located medial to the carotid sheath in the neutral position, remained in the same place with 15 degrees of rotation in five patients. These statistically significant (*p* < 0.001) data show that, compared to the neutral position, head rotation to the opposite side may create an obstacle in the surgical path due to the SCM muscle covering the carotid sheath. Thus, the surgical time and possibility of complications may change.

Anatomical variations in the vagal nerve are quite common. In addition, since it is a mobile structure, its localization can change, especially with head rotation. The vagal nerve in the cervical region is generally located within the carotid sheath, between the common or internal carotid artery and the internal jugular vein [11]. Branching patterns within the carotid sheath can be seen in 29% of patients [12]. There are positional variations with the carotid artery and with the internal jugular vein during the course of the vagus nerve. In a study by Planitzer et al. [8], the position of the vagus nerve in patients was viewed, and in 42%, it was dorsolateral; in 24%, it was lateral; in 14%, it was dorsal; in 9%, it was ventrolateral; in 6%, it was ventral; in 3%, it was medial; and in 2%, it was dorsomedial to the carotid artery. Additionally, in 64%, it was dorsomedial; in 23%, it was medial; in 9%, it was dorsal; in 2%, it was ventral; and in 2%, it was ventromedial to the internal jugular vein. It was stated that there was no significant difference in these positional variations when gender and the right/left vagal nerve were compared [8]. Takamizawa et al. [9] examined the vagus nerve position intraoperatively in patients who underwent VNS surgery. In 8 (10.9%) patients, it was medial to the internal jugular vein (IJV) and ventral to the common carotid artery (CCA) (type 1); in 4 (5.5%) patients, it was medial to the IJV and lateral to the CCA (type 2); in 51 (70%) patients, it was dorsomedial to the IJV and ventral to the CCA (type 3); in 7 (9.5%) patients, it was dorsomedial to the IJV and lateral to the CCA (type 4); and in 3 (4.1%) patients, it was dorsal to the CCA (type 5) [9]. Ahn et al., in a neck ultrasonography study in which they examined bilateral vagal nerve variations in 536 patients, reported that positional differences in the vagal nerve were relatively common, and the anteromiddle type was the most common type observed [13]. Despite all these studies on the localization of and variations in the vagal nerve, the localization of the vagus nerve according to the position of the head has not been examined in the literature. With the peroperative ultrasonography we used for patients who underwent vagal nerve stimulation surgery, we observed that the vagal nerve in neutral head position was most frequently between the carotid artery and jugular vein in 58.3% of patients, it was located behind one of these vascular structures in 33.3%, and it was located in front of one of these vascular structures in 8.3%. When the head was rotated 15 degrees to the opposite side, it was observed that the vagus nerve was mostly located between the carotid and jugular vein in 47.2% of patients, behind one of these vascular structures in 27.8%, and in front of one of these vascular structures in 25%. As a result of our study, it is understood that, similar to in the literature, the vagal nerve can have different localizations within the carotid sheath, depending on the head position or not. We think that it is important to determine the nerve localization when planning surgery for the vagal nerve in order to prevent possible complications.

The use of ultrasound in neurosurgery practice is not new. Its use is increasing in areas such as ventricular catheter placement, spinal cord tumor surgery, traumatic cerebral hematoma surgery, glioma resection, optic nerve diameter measurements in intensive care units, pediatric ventricle diameter measurements, and many other pathologies [14]. Technical features have been defined to visualize peripheral and cranial nerves in ultrasonography. Because the nerves are in the shape of a tube, they may not be easily distinguished on longitudinal scans. It is recommended to view them in the transverse plane. The appearance of the nerve on the transverse scan is quite characteristic and can easily be distinguished. Internal fascicles, which form the internal structure of the nerve, are visualized as bundles of hypoechoic fascicles interspersed within the hyperechoic fatty tissue. This is described as the “honeycomb” view. The blood within the vein is hypoechoic. Arterial structures can be distinguished very easily with the Doppler feature of ultrasound [15].

Imaging of the vagus nerve prior to VNS implantation may not be necessary for the majority of surgeons. Even if the vagus nerve has a normal structure, it should be kept in mind that in patients with enlarged thyroid or lymph tissue, there may be adjacent tissue pathologies that affect the carotid sheath and therefore the vagus position, aside from the normal positional variations of the vagus nerve. There may be exceptional cases for this reason. There have been intraoperative neuromonitoring studies conducted to reduce the risk of nerve damage due to the displacement and pushing of the vagus nerve associated with an enlarged thyroid [16]. Intraoperative findings of a massive hypertrophic vagus nerve were reported in a patient with neurofibromatosis type 1 (NF1) who had no preoperative findings of vagus nerve dysfunction [17]. Although rare, there are publications reporting NF-2-associated bilateral vagal nerve neurofibroma [18]. Peroperative cervical ultrasonography is an easily applicable imaging method used to exclude the possibility of an enlarged vagus nerve without any symptoms and lesions such as neurofibroma and schwannoma in patients with neurocutaneous disease or in syndromic patients. Ultrasonography studies frequently show vagal nerve atrophy in neurodegenerative diseases such as Bulbar ALS and Parkinson’s. Ultrasonography studies also show vagal nerve enlargement in Charcot–Marie–Tooth disease with epilepsy [19,20,21].

In a series of 314 patients for whom the cervical vagus nerve was examined bilaterally with transcutaneous sonography, it was stated that 626 (99.6%) lower cervical vagus nerves and 551 (87.7%) upper cervical vagus nerves could be identified, and cervical vagus could not be distinguished by computed tomography or magnetic resonance imaging [22]. In another study conducted to visualize the location of the vagal nerve to reduce complications associated with surgical manipulation, they used fused images of three-dimensional computed tomography angiography (3D-CTA) and magnetic resonance imaging (MRI) preoperatively in two patients. Images were merged using software (Zaiostation 2, Ziosoft, Tokyo, Japan), and the time to detection of the vagal nerve was <60 min in both cases. However, it was stated that motion artifacts may have occurred due to swallowing during MRI, radiation exposure due to 3D-CTA was important, and the cardiac branches of the vagal nerve could not be visualized [23]. Ultrasonography, with fewer side effects, not only shows the cervical vagus nerve but also demonstrates the adjacent abnormal lesion involving the nerve [24,25]. In addition, studies measuring increasing ultrasonographic vagal nerve diameter and the diagnostic value of these parameters in various diseases can be determined simultaneously with vagal nerve stimulation surgery [26]. Ultrasound can sometimes be preferred because it is a practical, low-cost imaging method that provides instant and real-time imaging, is relatively easy, can be applied to every patient, and helps with identifying possible additional pathologies of the cervical region. Moreover, the anatomical structure of the cervical region facilitates the use of ultrasonography in this region.

Surgical complications of VNS treatment can be divided into early and late periods. Among the complications related to the vagal nerve, bradycardia and asystole, which can be seen during the lead impedance test, vagal nerve injury, dyspnea, and dysphagia can be seen in the early period. Delayed arrhythmias (bradycardia, asystole), laryngopharyngeal dysfunction, and neuralgia may be observed in the late period. Laryngopharyngeal dysfunction (hoarseness, shortness of breath, and coughing) may develop if inferior (recurrent) laryngeal nerve injury occurs during the identification and dissection of the vagal nerve from the surrounding tissue phase. This may cause hoarseness due to direct nerve stimulation when leads are mistakenly applied to the inferior (recurrent) laryngeal nerve instead of the vagus nerve. Stimulations > 40 Hz may cause tetanic adduction with laryngeal hemispasm (1–2.7%). It has also been reported that drooling and hyperactivity may increase in children [7]. There are also some other risks like bleeding due to laceration of the carotid artery or internal jugular vein, cerebral ischemia due to its occlusion, or venous infraction. We believe that a short surgery time, a minimal and short retouching period, and surgical intervention of the vagus nerve in the appropriate position are important to reduce or prevent this broad spectrum of complications.

### Study Limitations

The primary limitation of this study is its retrospective design. Excluding patients for whom sufficient data could not be obtained reduced the sample size of the study. In anatomical studies related to the vagus nerve, the neck position has either not been specified or has been considered neutral. Since research on the change in anatomical position with cervical rotation movement, as examined in our study, has not been identified in the literature to our knowledge, the discussion on this topic was limited.

## 5. Conclusions

The SCM muscle covers the carotid sheath or increases the amount of closure with head rotation. The vagus nerve, however, does not show a regular positional change due to anatomical and positional variations, and it can be seen in different locations in each patient. Understanding the anatomy, especially the position of the nervus vagus, reduces the possibility of complications in vagal nerve stimulation surgery. Ultrasonography is fast, practical, reliable and applicable to every patient and provides real-time imaging. We recommend that peroperative ultrasonography be used routinely in vagal nerve surgery to determine an appropriate head position for the surgical route and vagus nerve localization.

## Figures and Tables

**Table 1:** Distribution of data by gender.

	Female (n:20)	Male (n:16)	*p*-Value
Age (mean ± SD)	32.25 ± 5.96	31.62 ± 5.13	0.629
SCM localization in neutral position, n (%)			0.487
Lateral to the carotid sheath	8 (22.2%)	8 (22.2%)	
Above the carotid sheath	10 (27.8%)	5 (13.9%)	
Medial to the carotid sheath	2 (5.6%)	3 (8.3%)	
SCM localization at 15-degree rotation, n (%)			0.736
Lateral to the carotid sheath	2 (5.6%)	3 (8.3%)	
Above the carotid sheath	9 (25%)	6 (16.7%)	
Medial of the carotid sheath	9 (25%)	7 (19.4%)	
N. Vagus localization in neutral position, n (%)			0.526
Anterior to JV-C	1 (2.8%)	2 (5.6%)	
Between JV-C	11 (30.6%)	10 (27.8%)	
Posterior to JV-C	8 (22.2%)	4 (11.1%)	
N. Vagus localization at 15-degree rotation, n (%)			0.598
Anterior to JV-C	6 (16.7%)	3 (8.3%)	
Between JV-C	8 (22.2%)	9 (25%)	
Posterior to JV-C	6 (16.7%)	4 (11.1%)	

SD: standard deviation; SCM: sternocleidomastoid muscle; JV-C: jugular vein–carotid artery.

**Table 2:** Localization change of sternocleidomastoid muscle according to the carotid sheath from neutral position to 15-degree rotation.

		15-Degree Rotation				
		Lateral to the Carotid Sheath n (%)	Above the Carotid Sheath n (%)	Medial to the Carotid Sheath n (%)	Total n (%)	
Neutral Position	Lateral to the Carotid Sheath n (%)	5 (13.9%)	11 (30.6%)	0	16 (44.4%)	*p* < 0.001
	Above the Carotid Sheath n (%)	0	4 (11.1%)	11 (30.6%)	15 (41.7%)	
	Medial to the Carotid Sheath n (%)	0	0	5 (13.9%)	5 (13.9%)	
	Total n (%)	5 (13.9%)	15 (41.7%)	16 (44.4%)	36 (100%)	

**Table 3:** Localization change of nervus vagus inside carotid sheath from neutral position to 15-degree rotation.

		15-Degree Rotation				
		Anterior of JV-C n (%)	Between JV-C n (%)	Posterior of JV-C n (%)	Total n (%)	
Neutral Position	Anterior of JV-C n (%)	2 (5.6%)	1 (2.8%)	0	3 (8.3%)	*p*:0.198
	Between JV-C n (%)	5 (13.9%)	10 (27.8%)	6 (16.7%)	21 (58.3%)	
	Posterior of JV-C n (%)	2 (5.6%)	6 (16.7%)	4 (11.1%)	12 (33.3%)	
	Total n (%)	9 (25%)	17 (47.2%)	10 (27.8%)	36 (100%)	

JV-C: jugular vein–carotid artery.

## Data Availability

Data are available to share only by request from the corresponding author because of ethical and privacy issues.

## Abbreviations

The following abbreviations are used in this manuscript:

SCM	Sternocleidomastoid
C	Carotid artery
JV	Jugular vein
